# Hybrid Swarming Algorithm With Van Der Waals Force

**DOI:** 10.3389/fbioe.2022.806177

**Published:** 2022-02-23

**Authors:** Zhang Yi, Yu Hongda, Sun Mengdi, Xu Yong

**Affiliations:** College of Electrical and Computer Science, Jilin Jianzhu University, Changchun, China

**Keywords:** van der waals forces, ant colony optimization, physarum polycephalum algorithm, swarming algorithm, hybrid

## Abstract

This paper proposes a hybrid swarming algorithm based on Ant Colony Optimization and Physarum Polycephalum Algorithm. And the Van Der Waals force is first applied to the pheromone update mechanism of the hybrid algorithm. The improved method can prevent premature convergence into the local optimal solution. Simulation results show the proposed approach has excellent in solving accuracy and convergence time. We also compare the improved algorithm with other advanced algorithms and the results show that our algorithm is more accurate than the literature algorithms. In addition, we use the capitals of 35 Asian countries as an example to verify the robustness and versatility of the hybrid algorithm.

## Introduction

Some problems in the real world can be converted into a Traveling Salesman Problem (TSP) such as Route Planning Problem, Goods Distribution Problem and Vehicle Scheduling Problem. Traveling Salesman Problem is a classic NP-hard problem. Traditional accurate algorithms are difficult or unable to solve NP-hard problem. The heuristic algorithms such as Ant Colony Optimization (Dorigo and Caro, 1999), Particle Swarm Optimization (Wang et al., 2003), Bat Algorithm (YANG, 2010), Pigeon Swarm Algorithm (Duan and Qiu, 2019) are all inspired by swarm intelligence and used to solve the NP-hard problem effectively. The heuristic algorithms generally appear to fall into local optima and slow convergence as the scale of the problem continues to expand (Bouzbita et al., 2018; de S. Alves et al., 2018). Many improved methods have been proposed to optimize traditional accurate applications. Li, X (Li and Yin, 2011) presents a new method of designing a reconfigurable antenna array with quantized phase excitations using a new evolutionary algorithm called differential evolution (DE). To reduce the effect of mutual coupling among the antenna-array elements, the dynamic range ratio is minimized. Cui et al. (2020) proposed a blockchain-based multi-WSN authentication scheme for the Internet of Things, which builds a blockchain network between different types of nodes to form a hybrid blockchain model and realizes mutual authentication of node identities in various communication scenarios. Cai et al. (2021) proposed a multi-objective optimization algorithm based on the dynamic reward and punishment mechanism to optimize the validity model of shard verification, which significantly improves the throughput and effectiveness of shards, thereby supporting blockchain-enabled. Zhang et al. (2021) designed a novel weight-based integrated machine learning algorithm (WBELA) to identify abnormal messages in the vehicle controller area network (CAN) bus network, and a multi-objective optimization algorithm based on balance convergence and diversity to improve the accuracy, reduce the false alarm rate, and improve the security of the 6G vehicle network. Li, X (Li and Ma, 2017) presents a novel multi-objective memetic search algorithm (MMSA), which is proposed to solve the MOPFSSP with makespan and total flowtime. Cui et al. (2021) designed a multi-objective constrained Boltzmann machine (RBM) model for training, used evaluation indexes to comprehensively measure the effect of data classification, introduced policy pool and FFT to improve the effectiveness of data fusion, and used a non-dominated sorting genetic algorithm (NSGA-II) to deal with unbalanced malware families. Xin (2015) proposed a genetic algorithm for this problem. An enhanced crossover strategy and three different local searches are adopted. After the exhaustive computational and statistical analysis, we can conclude that the proposed methods are robust and outperformed the existing algorithm.

Novel swarming algorithms emerge constantly in recent years. In this paper, we apply the Physarum Polycephalum Algorithm (PPA) in the path planning problem because it has good application results in routing problems. PPA is inspired by its behavior for its efficient ability to construct a foraging network path. Researchers have done much research on physarum polycephalum. Cifarelli et al. (2014) realized the adaptive network and the spatial distribution of nano- and micro-scale materials by embedding and programming different chemical substances in Phytophthora polycephalum and confirmed that the physarum network could be used as a scaffold. Develop hybrid nanocircuits, microcircuits and devices. Dimonte et al. (2015) load Physarum with magnetic particles and place them in a magnetic field, and in principle apply analog control programs to precisely control the active growth area of slime mold and the shape topology of its protoplasmic network, making it an invaluable substrate capable of designing novel sensing, computing, and driving architectures in biological substrates. Liu et al. (2018) According to Polycephalum can establish a biological network and distribute traffic according to the location and size of food sources; they proposed a fuzzy user balance model for urban traffic allocation based on Polycephalum Algorithm. The biological network relates to the transportation network. Tsompanas et al. (2016) improved the time efficiency of biological computers by using cellular automata (CA) and digital circuits and used the Greek topology as the input of the biological computer, which effectively improved the PPA computing power. Zhang et al. (2016) proposed a supply chain network design algorithm based on PPA and used numerical calculations to demonstrate the practicality and robustness of the modified Algorithm. Arslan and Manguoglu (2019) proposed a parallel PPA which proved the parallel scalability and accuracy of the algorithm by using the parallel iterative linear solver and the parallel proposition of the *M* matrix. A typical swarm algorithm has the advantage of fast solving speed, but its disadvantage is easy to fall into a locally optimal solution. In this paper, we introduce Van Der Waals Forces (VDWF) to improve this problem. VDWF is the forces between molecules. It is a weakly basic electrical attraction between neutral molecules or atoms. It is characterized by far absorption and near repulsion. VDWF has shown significant contributions to academic research as an intermolecular force. Oakland (Ouakad, 2017) analyzed the influence of VDWF on the mechanical performance of MEMS/NEMS actuators and developed a reduced-order model based on the original Galerkin expansion, which confirmed that the VDWF could capture the separation length of the actuator parameters. The ability to influence. Liu et al. (2021) studied the interaction of VDWF between stacked components, revealed the microscopy technique of the relationship between the structure and properties of VDWF heterostructures, and analyzed the spectroscopic measurement of the VDWF interface coupling effect.

In this paper, we present a hybrid swarming algorithm to improve performance. The algorithm mix Ant Colony Algorithm and Physarum Polycephalum Algorithm. Van Der Waals Force is introduced to prevent the algorithm from falling into the local optimal solution. The structure of this paper is as follows. In section 2, we introduce the basic concepts and formulas of the traveling salesman problem, Van Der Waals force, Ant Colony Algorithm, Physarum Polycephalum Algorithm. In section 3, We describe the framework of the improved hybrid Algorithm. In section 4, We provide the contrastive results of different algorithms for solving the TSP problem on the benchmark dataset. Moreover, we detail analysis of the data results. Section 5 concludes this paper.

## Related Work

Section 2.1 describes the Traveling Salesman Problem; section 2.2 introduces the Van Der Waals Forces; section 2.3 explains the ant colony algorithm. Finally, section 2.4 shows the Physarum Polycephalum model.

### Traveling Salesman Problem

Traveling Salesman Problem can be described as follows: a salesman is going to travel finally returns to the initial city. Its model is to find a travel route with the shortest total distance and satisfy the objective function:

$$L\left(C\right)=\min\sum_{i=1}^{n-1}d\left(c_{i},c_{i+1}\right)+d\left(c_{n},c1\right)$$ (1)

Where *c*_*i*_ is the city number i∈n , 1≤i≤n ; n is the number of cities; $d\left(c_{i},c_{j}\right)$ is the city i The length of the distance to the city j .

### Van Der Waals Forces

Van Der Waals Force is a weakly alkaline electrical attraction between neutral molecules or atoms. It comes from three parts: 1) One of the permanent dipole moments of polar molecules; 2) The interaction between a polar molecule polarizes another molecule, generating an induced dipole moment and attracting each other; 3) The movement of electrons in a molecule generates an instantaneous dipole moment, which makes neighboring molecules Instantaneous polarization, the latter in turn enhances the instantaneous dipole moment of the original molecule. Its formula is:

$$F=\frac{A}{d^{a}}-\frac{B}{d^{b}}$$ (2)

Where d is the distance between two molecules; A,B,a,b are all self-selected values, where a<b .

### Ant Colony Optimization

Ant Colony Optimization is a heuristic bionic algorithm proposed by Dorigo and Caro (1999). The principle is that when ants are looking for food, they will leave volatile pheromone on the path, and subsequent ants will tend to choose the path with high pheromone concentration. As time goes by, there will be more and more ants on the shortest path. At a particular moment, the transition probability of the ant choosing node *j* from node *i* is:

$$P_{ij}^{k}\left(t\right)=\{\begin{array}{cc} \frac{{\left[\tau_{ij}\left(t\right)\right]}^{\alpha }{\left[\eta_{ij}\left(t\right)\right]}^{\beta }}{\sum_{x\in s_{k}}{\left[\tau_{is}\left(t\right)\right]}^{\alpha }{\left[\eta_{is}\left(t\right)\right]}^{\beta }}, & j\in N_{k}\\ 0 & otherwise \end{array}$$ (3)

Where $\tau_{ij}t$ is the pheromone concentration on the path between node *i* and node *j*, $\eta_{ij}\left(t\right)$ is the heuristic information, the reciprocal of the distance between node *i* and node *j*, α and β are the critical factors of pheromone concentration and the vital factor of heuristic information respectively, and N_k is the set of optional nodes. The pheromone update rule for ants is:

$$\tau_{ij}\left(t+1\right)=\left(1-\rho \right)\tau_{ij}\left(t\right)+\sum_{k=1}^{m}{\left(L_{k}\right)}^{-1}$$ (4)

Where ρ(0≤ρ≤1 ) is the volatilization coefficient of the pheromone and $L_{k}$ is the length of the path traveled by the ant k  in this cycle.

### Physarum Polycephalum Algorithm

The food source is regarded as the node and the spreading hyphae. They composed of the pipe and the liquid flowing inside in the foraging formed by Physarum Polycephalum. The pressure difference between the two ends of the pipe determines the flow direction of the liquid. The pipe becomes thicker when the liquid flow rate increases. It will eventually form the shortest path connecting the food source at last. The liquid flow through the pipe can be expressed as:

$$Q_{ij}=\frac{\pi r_{ij}^{4}\left(P_{i}-P_{j}\right)}{8\xi L_{ij}}=\frac{D_{ij}}{L_{ij}}\left(P_{i}-P_{j}\right)$$ (5)

Where ξ is the viscosity coefficient of the liquid in the pipe, $Q_{ij}$ is the liquid flow between node i and node *j*, and $P_{ij}$ is node *i* and node *j* The pressure between nodes, $D_{\left(ij\right)}$ is the conductivity between node *i* and node *j*, $L_{\left(ij\right)}$ is the Euler distance between node *i* and node *j*, $r_{ij}$ is the radius of the tube. According to Kirchhoff’s law, the flow of liquid in the tube is conserved, so $I_{0}$ is a constant, and the flow of each node can be expressed as:

$$\sum_{j=1,j\neq i}^{n}Q_{ij}=\{\begin{array}{c} \begin{array}{cc} I_{0}, & \;j=in \end{array}\\ \begin{array}{cc} -I_{0}, & j=out; \end{array}\\ \begin{array}{cc} 0, & otherwise \end{array} \end{array}$$ (6)

The change in conductivity over time is expressed as follows:

$$\frac{dD_{ij}}{dt}=f\left(\left|Q_{ij}\right|\right)-kD_{ij}$$ (7)

Where *k* is the attenuation rate of the pipeline.

## Improved Hybrid Algorithm Based on Van Der Waals Forces

The feature of ACO is the positive feedback relationship between the number of ant colonies and pheromone concentration. This also causes ACO easy to fall into local optimality. Moreover, many parameters also limit its optimization ability. These factors make ACO unable to be effective. The traditional ACO is low efficiencies in large-scale problems. This paper proposes a hybrid algorithm based on Van Der Waals Force (VPACO). This section focuses on the comparison of improved algorithms.

### A Hybrid Algorithm Based on Physarum Polycephalum Algorithm and Ant Colony Optimization

The pheromone update mechanism of the PACO algorithm is as fellow based on formula 4.

$$\tau_{ij}\left(t+1\right)=\left(1-\rho \right)\tau_{ij}\left(t\right)+\sum_{k=1}^{m}{\left(L_{k}\right)}^{-1}+\lambda e^{-{\frac{t}{T_{\max}}}^{2}}\frac{Q_{ij}M}{I_{0}}$$ (8)

Where λ (0 ≤λ≤1) is any constant, *t is* the current iteration number, $T_{max}$ is the maximum number of iterations, *M* is the square of the number of cities, $M=n^{2}$ . In Liu et al. (2017), authors present a hybrid algorithm named PACO to solve TSP. The pseudo code shows as Algorithm 1.

Algorithm 1 PACO pseudo code for solving TSP problem.

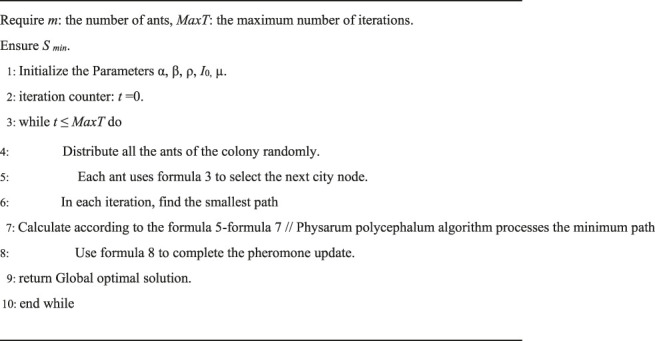

### A Hybrid Algorithm Based on Van Der Waals Force

In this paper, our improvement is named VPACO that the pheromone updating mechanism is optimized by adding Van Der Waals force. Formula 8 shows the method of our improvement. The pseudo code of VPACO shows in Algorithm 2.

$$\tau_{ij}\left(t+1\right)=\left(1-\rho \right)\tau_{ij}\left(t\right)+\sum_{k=1}^{m}{\left(L_{k}\right)}^{-1}+\lambda e^{-{\frac{t}{T_{\max}}}^{2}}\frac{Q_{ij}M}{I_{0}}+\frac{A}{d_{ij}^{a}}-\frac{B}{d_{ij}^{b}}$$ (9)

In our improvement, we set parameters A = 100, B = 100, a = 1.0, and b = 1.1.

Algorithm 2 VPACO pseudo code for solving TSP problem.

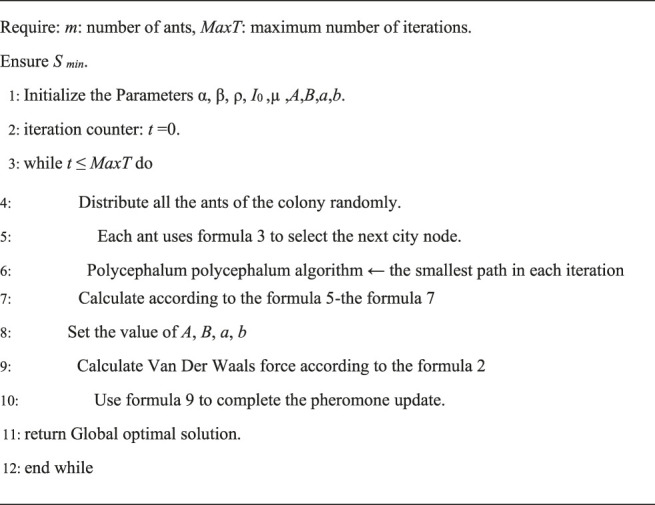

The improved Algorithm shows that VPACO can enhance the optimization ability. It can find a better-expected value and speed up the convergence. Moreover, the optimal solution can be found in a shorter time. Experiments show that VPACO can solve the classic TSP with large-scale problems.

## Experimental Research and Analysis

This section describes the parameter settings, analyzes the test results. Compares the best results by solving the same test for VPACO with PACO and ACO. Finally, to test the adaptability of VPACO, we apply it to real-world geographic travel problems. The experiment results are using Intel CoreTMi5-5200U CPU @ 2.20 GHz 6, 00 GO RAM and Windows 10, 64-bit operating system, x64-based processor. VPACO, PACO, and ACO design by python language. The test data come from http://comopt.ifi.uni-heidelberg.de/software/TSPLIB95/. For each instance, VPACO, PACO, ACO are implemented 10 times, and each iteration ends 500 times.

### Parameter Settings

The parameter adjustment of the Algorithm is significant for its convergence and whether it can find the optimal solution. In this experiment, we recommend setting the parameters of the running Algorithm according to a variety of configurations. After a series of runs, the parameter values of the three algorithms are shown in Table 1, where *m* represents the number of ants.

**TABLE 1:** Parameter initialization.

Instance	*m*	Α	β	ρ	µ	*I*_0_
ch130	50	0.50	4.05	0.9	0.5	80
kroA100	40	0.50	4.05	0.9	0.5	220
kroB100	40	0.50	4.05	0.9	0.5	200
kroD100	33	0.50	4.05	0.9	0.5	190
eil101	35	0.50	4.05	0.9	0.5	22

### Experimental Results and Discussion

First, we provide the current best performance comparison of VPACO, PACO, and ACO calculation results on five instances in the TSPLIB benchmark set, as shown in Table 2. In each instance, the calculation result is composed of $S_{\min}$ , $S_{average}$ , $S_{\mathrm{variance}}$ and Gap . Among them, the data of *S_min_* in the table is shown the minimum value of the instance and *S_average_* is shown the average value of the instance. The *Alg* represents the three algorithms we used for comparison. $S_{\mathrm{variance}}=\sqrt{\frac{\sum_{i=1}^{500}{\left(S_{i}-\bar{S}\right)}^{2}}{500}}$ , $Gap=\frac{S_{\min}-S_{optimal}}{S_{optimal}}\times 100\%$ . The'Optimal’ column in the table, the data comes from http://comopt.ifi.uni-heidelberg.de/software/TSPLIB95/STSP.html. The Gap can be used to measure the deviation rate between the experimental minimum and the optimal solution. The smaller the value of *Gap*, the higher probability that the algorithm obtains the optimal solution in each run. It can be seen from the table that the Gap value of VPACO is always the smallest. Table 2 also shows that the various calculation results of VPACO are better than PACO and ACO, which proves that the optimization ability of VPACO is more efficient than that of PACO and ACO. At the same time, the optimization ability of PACO is also efficient than that of ACO. $AVR=\left|\frac{S_{average}-S_{optimal}}{S_{optimal}}\right|\times 100\%$ is used to measure the deviation rate between the average and the best value. VPACO has the smallest *AVR* value compared with PACO and ACO. The *AVR* difference between VPACO and PACO is less than two, and the *AVR* difference between PACO and ACO is vast.

**TABLE 2:** The comparison results of VPACO, PACO, ACO on the instances.

Instance	Optimal	Alg	$S_{\min}$	$S_{average}$	$S_{\mathrm{var}iance}$	Gap	AVR
Ch130	6,110	VPACO	6,150.9	6,219.3	188.8	**0.65**	**1.79**
		PACO	6,198.2	6,273.45	196.45	1.44	2.68
		ACO	6,778.0	6,876.4	566.4	10.9	12.54
Eil101	629	VPACO	642.7	650.4	21.1	**2.06**	**3.40**
		PACO	674.2	654.4	21.5	7.15	4.04
		ACO	705.4	769.1	69.7	12.1	22.27
KroA100	21,282	VPACO	21,298.2	21,611.7	752.8	**0.07**	**1.55**
		PACO	21,663.4	22,203.3	1,475.3	1.79	4.33
		ACO	23,198.5	24,540.1	1,609.1	9.00	15.31
KroB100	22,141	VPACO	22,277.0	22,521.9	779.7	**0.61**	**1.72**
		PACO	22,597.5	23,176.4	1,164.7	2.06	4.67
		ACO	24,659.4	25,672.1	1,417.7	11.37	15.95
KroD100	21,295	VPACO	21,309.6	21,453.9	526.1	**0.07**	**0.74**
		PACO	21,764.5	22,294.6	1,267.9	2.20	4.69
		ACO	22,559.0	23,829.4	1,493.4	5.94	11.90

**FIGURE 1:**
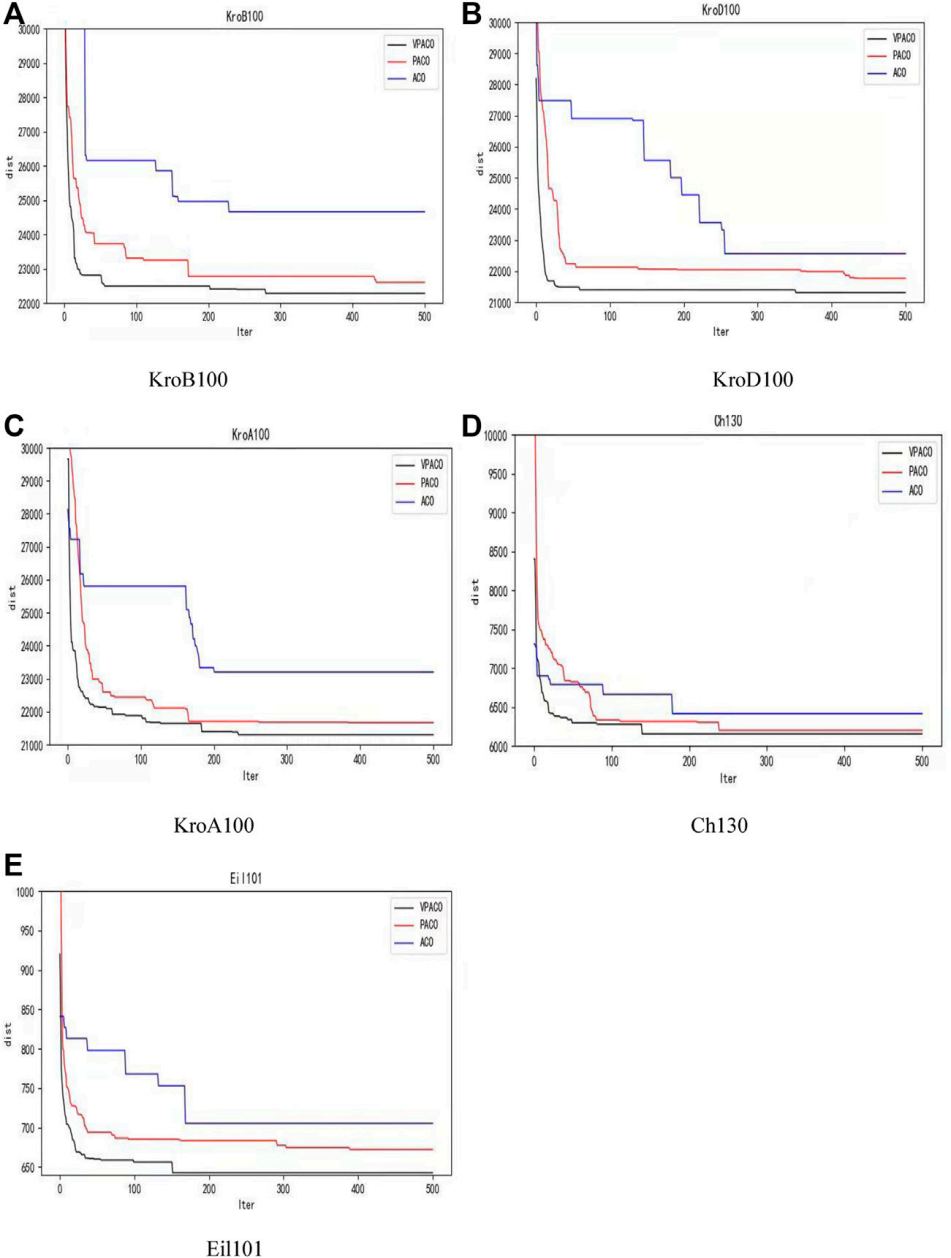
The change curve of the optimization result with the number of iterations in TSPLIB instances: **(A)** KorB100, **(B)** KorD100, **(C)** KorA100, **(D)** Ch130, **(E)** Eil101.

Figure 1 shows the convergence curve of the optimization results in each instance with the number of iterations. The gap between the three algorithms is not apparent in the previous iterations. The convergence and optimization ability of VPACO and PACO is far better than ACO as the number of iterations increases. The convergence of VPACO is the same as PACO, but VPACO’s optimization ability is better. Applying Van Der Waals Force to the Algorithm is effective for the optimization ability.

Finally, we compare VPACO algorithm and other improved algorithms based on ACO with the simulation data set in TSPLIB. In Table 3, Opt show the best result in TSPLIB, and VPACO show our best results, MMAS show the results according to Pang et al. (2016), ACADCG show the results according to Sun et al. (2004), IGSA show the results according to Zhang and Yuxian (2017) IGA show the results according to Zhang et al. (2016). It is obviously that the optimal solution obtained by VPACO algorithm are basically consistent with the official optimal value given in TSPLIB. The VPACO algorithm is more accurate, stable, and reliable compared with other mainstream algorithms mentioned in this paper.

**TABLE 3:** Test results of VPACO compared with the improved algorithms.

Test	Opt	VPACO	MMAS	ACADCG	IGSA	IGA
Eil51	426	**426**	436.63	428.19	460	428.87
St70	675	**675**	685.13	681.25	—	—
Eil76	538	**538**	552.26	543.43	548	544.37
Oliver30	423	**423.74**	424.86	423.74	—	—

### Real World Geographic Travel

To verify the effectiveness of our improved Algorithm in the natural environment, we considered a real scenario: how to travel around 34 capitals in Asia with the lowest cost. The geographic coordinates are taken from http://api. map. baidu.com/lbsapi/getpoint/index.html shown in Table 4. The latitude is positive with north latitude, longitude with east longitude is positive. This problem can be regarded as TSP. We use the spherical distance calculation of the distance between cities, which can be calculated according to formula 10. Among them, $lat_{i}$ , $lon_{i}$ represents the latitude and longitude of the city *i* respectively, and $R_{0}$ represents the earth’s radius. This article takes $R_{0}=6378.137$ .

$$X1=\frac{lat_{A}\times \pi }{180}Y1=\frac{lon_{A}\times \pi }{180}X2=\frac{lat_{B}\times \pi }{180}Y2=\frac{lon_{B}\times \pi }{180}$$

$$D=2R_{0}\arcsin\sqrt{\sin^{2}\left(\frac{X1-X2}{2}\right)+\cos X1\cos X2\sin^{2}\left(\frac{Y1-Y2}{2}\right)}$$ (10)

**TABLE 4:** The geographical coordinates of capitals of 35 countries in asia.

No.	City	Latitude	Longitude	No.	City	Latitude	Longitude
1	Beijing	39.904	116.407	19	Tehran	34.528	69.172
2	Tokyo	35.673	139.758	20	Kabul	25.280	51.522
3	Ulaanbaatar	47.921	106.906	21	Ad Dawhah	26.217	50.583
4	HaNoi	21.033	105.850	22	Al Manamah	24.467	54.367
5	Vientiane	17.963	102.614	23	Abu Dhabi	24.712	46.724
6	PhnomPenh	11.559	104.917	24	Ar Riyad	23.614	58.591
7	Yangon	16.8	96.15	25	Masqat	15.352	44.207
8	Bangkok	13.820	100.665	26	Sana	33.332	44.418
9	Kuala Lumpur	3.139	101.687	27	Baghdad	25.407	55.433
10	Bandar Seri Begawan	4.890	114.942	28	Amman	33.516	36.314
11	Jakarta	−6.212	106.845	29	Dimashq	39.921	32.854
12	Dili	−8.558	125.578	30	Ankara	40.183	44.517
13	Manila	14.599	120.984	31	Erevan	41.710	44.793
14	Kathmandu	27.703	85.318	32	Tbilisi	40.435	49.868
15	Thimbu	27.467	89.642	33	Baku	41.267	69.217
16	Dhaka	23.710	90.407	34	Taskent	37.950	58.380
17	Islamabad	33.718	73.061	35	Ashabad	42.870	74.588
18	Colombo	6.927	79.861				

We use three methods to compute the shortest distance cost of travel. Both VPACO and PACO can find the minimum value, $S_{\min}=37168.12$ , but the value of S _variance_ of VPACO is smaller than PACO and ACO. The results shows that VPACO has more stability than PACO.

**FIGURE 2:**
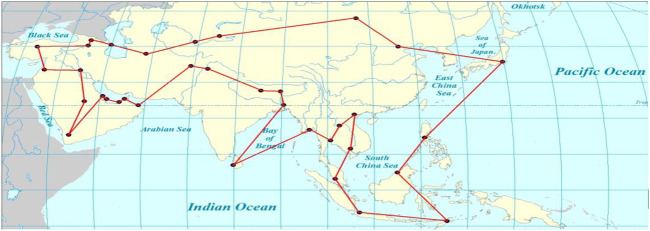
VPACO’s tourist route map of 35 Asian capitals.

Figure 2 shows the VPACO’s lowest distance cost tour route map, and better route planning.

## Conclusion

Optimization problems can be divided into discrete problems and persistent problems. VPACO is an improved heuristic algorithm for solving discrete problems. The improvement adds Van Der Waals Force to modify the pheromone update rule at the first time. It can enhance convergence and optimization ability. Simulation results have shown that the proposed approach has a higher coverage rate, more uniform configuration, and average convergence time are better than PACO and ACO. In addition, taking the capitals of 35 Asian countries as an example, the robustness and versatility of VPACO are once again verified. The improved method may be used to solve large-scale problems in the future.

## Data Availability

The original contributions presented in the study are included in the article/Supplementary Material, further inquiries can be directed to the corresponding author.
